# Remote ischemic preconditioning for prevention of contrast-associated acute kidney injury following percutaneous coronary intervention: a randomized controlled trial

**DOI:** 10.1093/ckj/sfaf342

**Published:** 2025-11-07

**Authors:** Ganesh Paramasivam, Ashika Bangera, Ashwija Kolakemar, Shravana Acharya, Ravindra Maradi, Shankar Prasad Nagaraju, Padmakumar Ramachandran, Tom Devasia, Attur Ravindra Prabhu, Indu Ramachandra Rao

**Affiliations:** Department of Cardiology, Kasturba Medical College, Manipal, Manipal Academy of Higher Education, Manipal, Karnataka, India; Department of Nephrology, Kasturba Medical College, Manipal, Manipal Academy of Higher Education, Manipal, Karnataka, India; Department of Nephrology, Kasturba Medical College, Manipal, Manipal Academy of Higher Education, Manipal, Karnataka, India; Department of Nephrology, Kasturba Medical College, Manipal, Manipal Academy of Higher Education, Manipal, Karnataka, India; Department of Biochemistry, Kasturba Medical College, Manipal, Manipal Academy of Higher Education, Manipal, Karnataka, India; Department of Nephrology, Kasturba Medical College, Manipal, Manipal Academy of Higher Education, Manipal, Karnataka, India; Department of Cardiology, Kasturba Medical College, Manipal, Manipal Academy of Higher Education, Manipal, Karnataka, India; Department of Cardiology, Kasturba Medical College, Manipal, Manipal Academy of Higher Education, Manipal, Karnataka, India; Department of Nephrology, Kasturba Medical College, Manipal, Manipal Academy of Higher Education, Manipal, Karnataka, India; Department of Nephrology, Kasturba Medical College, Manipal, Manipal Academy of Higher Education, Manipal, Karnataka, India

**Keywords:** acute kidney injury, contrast media, ischemic preconditioning, percutaneous coronary intervention, radiocontrast agents

## Abstract

**Background:**

Contrast-associated acute kidney injury (CA-AKI) has been reported to occur in a significant proportion of patients undergoing percutaneous coronary intervention (PCI). The role of remote ischemic preconditioning (RIPC) in CA-AKI prevention remains unclear.

**Methods:**

In this single-center double-blind randomized controlled trial, 420 eligible patients with high risk of CA-AKI admitted for PCI were randomized into two groups: RIPC and sham RIPC (control group). RIPC was performed by repeated cycles of inflation and deflation of an upper limb blood pressure cuff prior to PCI. Serum neutrophil gelatinase-associated lipocalin (NGAL) levels were measured at 2- and 6-h, and serum creatinine at 24- and 48-h post-PCI. The primary endpoint was the incidence of CA-AKI. Secondary endpoints were change in serum creatinine at 48 h, incidence of subclinical AKI (defined as an increase in NGAL values by 25% or more from baseline), change in NGAL at 2- and 6-h (delta NGAL), in-hospital mortality and major adverse cardiovascular events (MACE) at Day 30.

**Results:**

CA-AKI occurred in 11.4% (*n* = 48), with AKI stage 1 in 10.2% (*n* = 42). The incidence of CA-AKI was significantly lower in the RIPC group, compared with control group [8.1% vs 15.0%, risk ratio (RR) 0.54, 95% confidence interval (CI) 0.31–0.94; *P* = .027]. There was no significant difference in change in creatinine at 48 h in both the groups (*P* = .158). The incidence of subclinical AKI was also numerically lower in the RIPC group; however, this was not statistically significant (36.2% vs 40.5%, RR 0.89, 95% CI 0.66–1.22; *P* = .158). Dialysis-requiring AKI, in-hospital mortality and 30-day MACE were similar in both groups. There were no RIPC-related adverse events.

**Conclusions:**

In patients at high risk of developing CA-AKI after PCI, RIPC is an effective and safe preventive measure.

## GRAPHICAL ABSTRACT

**GRAPHICAL ABSTRACT:**
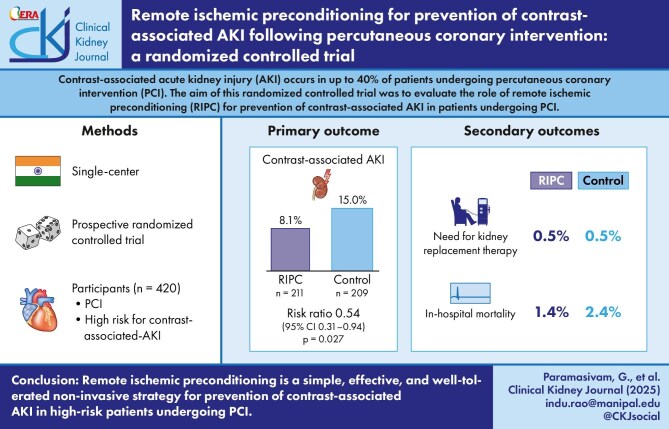

KEY LEARNING POINTS **What was known:**

- Patients receiving intravascular (especially intraarterial) iodinated contrast agents are at risk of developing contrast-associated acute kidney injury (CA-AKI).
- No specific treatment is available, and prevention remains the mainstay.
- Evidence on the role of remote ischemic preconditioning (RIPC) for CA-AKI prevention is conflicting.

**This study adds:**

- This study enrolled a percutaneous coronary intervention (PCI) cohort at high risk of CA-AKI, identified by Mehran score and serum creatinine/estimated glomerular filtration rate criteria.
- RIPC reduced CA-AKI risk by 46%, though no significant reduction was seen in dialysis-requiring AKI, in-hospital mortality or 30-day MACE.
- RIPC was safe, with no procedure-related adverse events.
- CA-AKI incidence was 11.4% in the cohort, with ∼90% being stage 1 AKI, suggesting that CA-AKI risk in contemporary practice is lower than historically reported.

**Potential impact:**

- This study suggests a benefit of RIPC for CA-AKI prevention and demonstrates that it could be safely applied even in the primary PCI setting.
- It also highlights that the occurrence of dialysis-requiring AKI following contrast exposure is rare, and therefore, life-saving procedures such as PCI should not be delayed due to the fear of AKI.

## INTRODUCTION

Contrast-associated acute kidney injury (CA-AKI) is a well-described complication that occurs due to intravascular administration of iodinated contrast agents. The incidence of CA-AKI is reported to vary from <1% to as high as 40%, and depends on the route of contrast administration (higher risk with intraarterial administration than with the intravenous route), clinical setting (higher risk in patients with ST elevation myocardial infarction), type of procedure (highest risk with percutaneous coronary intervention), and presence of risk factors such as older age, kidney disease, diabetes mellitus and dehydration [1]. The occurrence of CA-AKI has important clinical implications, including prolonged hospitalization, requirement of dialysis and increased mortality risk [2–4].

Since there is no specific treatment available, prevention is key. Of the various preventive strategies that have been tried, intravenous hydration with normal saline remains the cornerstone and is the only method consistently recommended by existing guidelines [5]. Unfortunately, despite widespread implementation of these measures, the global incidence of CA-AKI has remained unchanged [1]. Furthermore, hydration needs to be started 3–12 h prior to the procedure and is, therefore, not feasible in patients needing primary percutaneous coronary intervention (PCI), an emergency procedure for those with acute ST-elevation myocardial infarction (STEMI) [6, 7]. Aggressive hydration may also lead to acute pulmonary edema in some patients, especially in those with poor left ventricular function. As a result, there remains an unmet need for other effective methods for CA-AKI prevention.

Remote ischemic preconditioning (RIPC) refers to brief, repetitive periods of ischemia applied to a remote vascular bed to confer protection against prolonged ischemia in a distant (target) organ [8, 9]. It involves inducing transient limb ischemia by alternating cycles of inflation and deflation of a blood pressure (BP) cuff tied around the arm or leg. This non-invasive, non-pharmacological, safe and cost-effective technique is a promising strategy for CA-AKI prevention in patients undergoing coronary angiography and/or PCI. Even though the use of RIPC has shown encouraging results in a few studies, its role in the prevention of CA-AKI is still unclear [10–14]. There is also limited evidence for RIPC in the setting of primary PCI.

The aim of this randomized controlled trial (RCT) was to evaluate the role of RIPC for prevention of CA-AKI in patients undergoing PCI (either elective or primary) and at a high risk of developing CA-AKI (based on criteria from published literature) [3, 15–18]. We also sought to study the effect of RIPC on the incidence of subclinical AKI.

## MATERIALS AND METHODS

### Study design

This was a parallel group, double-blinded, sham-controlled RCT conducted at Kasturba Medical College and Hospital, Manipal, a tertiary-care hospital in South India between 1 March 2022 and 30 October 2024. Institutional ethics committee clearance was obtained (IEC 751–2019), and the study was registered in the Clinical Trials Registry of India (CTRI) (CTRI/2020/07/026 548). The trial was funded by an extramural research grant from the Indian Council of Medical Research (ICMR) and was conducted in accordance with the principles of the Declaration of Helsinki and the Good Clinical Practice guidelines. All included patients provided written informed consent.

### Study participants

Adults (18 years or older) of any gender admitted for either elective or primary PCI and considered to be at high risk for CA-AKI were eligible for inclusion. High risk for CA-AKI was defined by the presence of any one of the following:

(i) serum creatinine of >1.4 mg/dL or an estimated glomerular filtration rate (eGFR) of <60 mL/min/1.73 m^2^ [5, 19];
(ii) pre-PCI Mehran score ≥11 [16];
(iii) pre-PCI Mehran 2.0 score ≥8 [17].

The incidence of CA-AKI in patients with any of the above factors is expected to be >15%–20% [5, 16, 17, 20]. Although the original Mehran score (Mehran 1.0) is among the most widely used risk prediction tools for CA-AKI, it was primarily developed as a post-procedural score, incorporating variables such as contrast volume and hemodynamic status after PCI [16]. In this study, only pre-procedural variables from the original score were used to facilitate risk-stratification prior to PCI and determine eligibility for the study. In January 2023, the eligibility criteria were expanded to include the Mehran 2.0 risk score criterion to address slow recruitment and improve applicability to contemporary clinical populations, including patients with acute myocardial infarction. The Mehran 2.0 model consists of two versions—one based solely on pre-procedural variables and another incorporating both pre- and post-procedural variables [17]. For the purposes of this trial, only the pre-procedural version was used. This protocol amendment was prospectively approved by the Institutional Ethics Committee, and no patients were included retrospectively.

Exclusion criteria were patients on hemodialysis or peritoneal dialysis, recent intravascular contrast exposure (≤2 weeks prior to present admission), inability to perform RIPC due to any cause (e.g. bilateral upper limb trauma/amputation, severe ischemia, etc.), pregnancy, hypotension (mean arterial pressure <60 mmHg) and active sepsis.

### Randomization and blinding

Eligible inpatients scheduled to undergo PCI as part of standard care were assessed for eligibility. After obtaining written informed consent, participants were randomized in a 1:1 ratio into one of two groups, RIPC group or sham conditioning (control) group. Randomization was done using block randomization method with random block sizes (varying block sizes of four, six and eight) using computer-generated sequence. The Sealed Envelope online randomization tool (www.sealedenvelope.com) was used for sequence generation [21]. Allocation concealment was done using sequentially numbered, opaque, sealed envelopes, which were prepared by a member of the research team not involved in participant recruitment or outcome assessment. Envelopes were opened only after the participant had consented and was enrolled in the study. The study participants, clinical staff involved in patient care and follow-up (including cardiologists performing the PCI), and outcome assessors were blinded to the treatment allocation.

### Study intervention

All study participants received standard CA-AKI preventive measures. Intravenous hydration was administered as per departmental protocol, with the rate and timing of administration individualized based on the clinical context. In general, patients undergoing elective PCI received normal saline at 1–1.5 mL/kg/h, initiated 6–12 h prior to the procedure and continued for 12–24 h post-procedure. In those with severe left ventricular dysfunction, the infusion rate was reduced to 0.5 mL/kg/h to minimize the risk of volume overload. For patients undergoing primary PCI, hydration was initiated at a rate of 1.5–3 mL/kg/h upon arrival and continued at 1 mL/kg/h for 12–24 h post-procedure, unless contraindicated. While normal saline was the standard fluid, the use of sodium bicarbonate and adjunctive measures such as N-acetylcysteine (NAC) was permitted at the discretion of the treating physician. In addition, metformin and angiotensin-converting enzyme inhibitors or angiotensin receptor blockers were withheld for at least 24–48 h prior to the procedure in patients undergoing elective PCI and resumed 48 h after the procedure, depending on post-procedural renal function.

Participants in the RIPC group underwent four alternating cycles of 5-min inflation of a standard upper arm BP cuff to 200 mmHg (or 40 mmHg above the individual’s systolic BP, if the systolic BP exceeded 160 mmHg), followed by 5-min deflation to induce transient arm ischemia and reperfusion. Those in the control group underwent a sham RIPC procedure, consisting of four alternating cycles of 5-min inflation to 10 mmHg below the individual’s diastolic BP, followed by 5-min deflation. In both groups, the procedure was performed immediately prior to PCI by two trained research assistants, ensuring that the patient is unable to read the BP cuff inflation pressure applied in order to maintain blinding. In the event of unforeseen delays in PCI, additional cycles were administered, thereby ensuring that the time interval between the completion of the last RIPC cycle and contrast administration remained <45 min. The level of pain during RIPC was assessed using the numerical rating scale (NRS) on a scale of 0 to 10, with 10 indicating the highest degree of pain. Following the study intervention, PCI was performed as per standard practice using iso-osmolar contrast agent (iodixanol).

### Data collection and follow-up

Baseline clinical and laboratory data were collected using a standardized study proforma. Details of the PCI procedure, including the volume of iodixanol used, were noted. Blood samples for neutrophil gelatinase-associated lipocalin (NGAL) were collected at baseline and at 2- and 6-h post-PCI, centrifuged at 3000*g* for 10 min, serum was isolated and stored in aliquots at –80°C for batch analysis. NGAL assay in the serum samples was performed by latex immunoturbidimetric method using the Roche Cobas® 8000 modular analyzer. Serum creatinine was measured in mg/dL at 24- and 48-h post-PCI using an isotope dilution mass spectrometry-traceable kinetic Jaffe method and eGFR was calculated using the 2009 Chronic Kidney Disease Epidemiology Collaboration formula [22]. All recruited participants were followed for 30 days after hospital discharge.

### Study outcome

The primary outcome of the study was the incidence of CA-AKI, defined as an increase in serum creatinine by ≥0.3 mg/dL or an increase by ≥1.5 times baseline values within 48 h of PCI. The secondary outcomes included: (i) incidence of subclinical AKI, defined as serum NGAL increase by 25% or more from baseline (in those without CA-AKI) [23, 24]; (ii) change in serum creatinine from baseline to 48 h post-PCI (delta creatinine); (iii) change in serum NGAL from baseline to 2- and 6-h post-PCI (delta NGAL); (iv) need for renal replacement therapy; (v) in-hospital mortality; and (vi) major adverse cardiovascular events (a composite of cardiovascular death, non-fatal stroke, non-fatal MI or re-hospitalization for heart failure) at 30-day follow-up (30-day MACE).

### Sample size calculation and statistical methods

Assuming a CIN incidence of 30% in the control group and 18% in the intervention arm based on existing studies, with an alpha error of 0.05 and a power of 80%, the required sample size was 400 (200 subjects in each arm). Assuming that 5% cases may be lost to follow-up, we planned to enroll at least 420 patients (210 in each arm).

All statistical analyses were performed with SPSS 20.0. Mean and standard deviations are used to describe normally distributed continuous variables, while those that were not normally distributed are described as median and 25th and 75th percentiles. Categorical variables are expressed as frequencies and percentages. Chi-square test (or Fisher’s exact test) and Student’s *t*-test were used to compare categorical variables and normally distributed continuous variables, respectively, in the RIPC and sham conditioning arms. For comparison of continuous data that was not normally distributed, Mann–Whitney U test was used. Risk ratios (RR) along with 95% confidence intervals (CI) were computed to compare the risk of CA-AKI (and other outcome measures) in both groups. A *P*-value of <.05 was considered statistically significant.

## RESULTS

**Figure 1:**
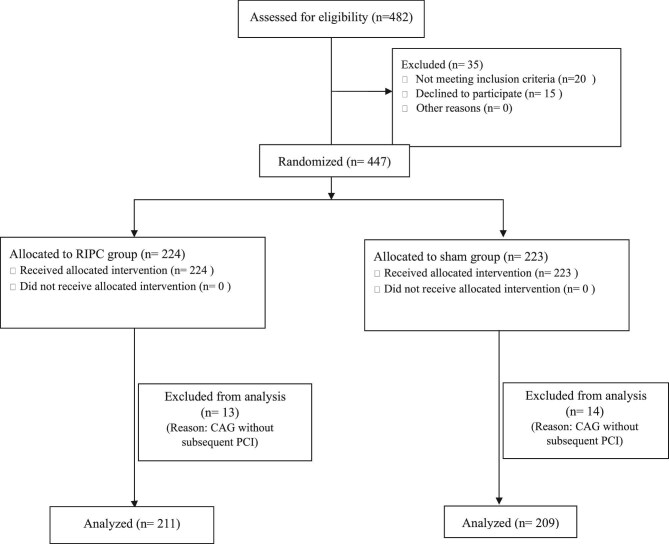
CONSORT flow diagram.

A total of 482 patients admitted for PCI during the study period were screened for eligibility. Of these, 447 participants were randomized after obtaining informed consent. After excluding those who underwent CAG without subsequent PCI, a total of 420 patients were included in the final analysis. The CONSORT (Consolidated Standards of Reporting Trials) flow diagram is presented below (Fig. 1). The baseline characteristics of the study participants are presented in Table 1. There were no significant differences between the two groups in age, gender, indication for PCI, comorbid illnesses or medications. The median serum creatinine (1.2 vs 1.3 mg/dL; *P* = .081) and eGFR were also comparable in both groups (57 vs 54 mL/min/1.73 m^2^, *P* = .126, respectively), as were other laboratory parameters. Both groups received similar measures for CA-AKI prophylaxis.

**Table 1:** Baseline characteristics of the study participants.

	Total cohort (*N* = 420)	RIPC group (*n* = 211)	Sham conditioning group (*n* = 209)	*P*-value
Demographic profile				
Age, years	64 (58, 72)	65 (58, 72.5)	63 (57, 72)	.648
Male, *n* (%)	298 (71.0)	148 (70.1)	150 (71.8)	.713
Diabetes mellitus, *n* (%)	272 (64.8)	128 (60.7)	144 (68.9)	.077
Hypertension, *n* (%)	264 (62.9)	132 (62.6)	132 (63.2)	.899
Dyslipidemia, *n* (%)	95 (22.6)	53 (25.1)	42 (20.1)	.219
Smoking, *n* (%)	61 (14.5)	35 (16.6)	26 (12.4)	.228
Previous PCI/CABG, *n* (%)	63 (15)	27 (12.8)	36 (17.2)	.204
Vitals and anthropometry				
SBP, mmHg	130 (120, 140)	130 (120, 140)	130 (120, 150)	.366
DBP, mmHg	80 (70, 90)	80 (70, 90)	80 (70, 90)	.456
BMI, kg/m^2^	24.9 (23.0, 26.6)	24.9 (22.6, 26.5)	25.0 (23.2, 26.6)	.374
Clinical presentation				.707
STEMI, *n* (%)	262 (62.4)	129 (61.1)	133 (63.6)	
NSTEMI, *n* (%)	102 (24.3)	49 (23.2)	53 (25.4)	
Unstable angina, *n* (%)	39 (9.3)	23 (10.9)	16 (7.7)	
Stable angina, *n* (%)	4 (1.0)	2 (0.9)	2 (1.0)	
Others, *n* (%)	13 (3.1)	8 (3.8)	5 (2.4)	
Laboratory values				
Hb, g/dL	12.8 (11.2, 14.3)	12.9 (11.1, 14.6)	12.6 (11.2, 14.2)	.562
HbA1c, %	6.5 (5.7, 8.3)	6.4 (5.7, 8.0)	6.7 (5.8, 8.4)	.172
Serum creatinine, mg/dL	1.3 (1.0, 1.5)	1.2 (1.0, 1.5)	1.3 (1.0, 1.5)	.081
eGFR, mL/min/1.73 m^2^	55 (44.0, 75.3)	57 (46.5, 78.5)	54 (42.0, 71.0)	.126
Albumin, g/dL	4.2 (3.9, 4.5)	4.2 (3.91, 4.5)	4.2 (3.8, 4.5)	.271
Serum NGAL, ng/mL^a^	96.4 (58.3, 146.0)	88 (58.0, 132.0)	103 (58.5, 148.0)	.281
Medications				
ACEi/ARB, *n* (%)	27 (6.4)	14 (6.6)	13 (6.3)	.872
SGLT2i, *n* (%)	83 (20.8)	38 (19.1)	45 (22.5)	.402
Insulin, *n* (%)	44 (10.5)	24 (11.4)	20 (9.6)	.546
Statin, *n* (%)	399 (95.0)	200 (94.8)	199 (95.2)	1.000
Diuretics, *n* (%)	154 (36.7)	76 (36.0)	78 (37.3)	.782
CA-AKI prophylaxis				
Saline only, *n* (%)	313 (74.5)	156 (73.9)	157 (73.1)	.747
Saline and sodium bicarbonate, *n* (%)	52 (12.4)	28 (13.3)	24 (11.5)	
Saline and NAC, *n* (%)	29 (6.9)	16 (7.6)	13 (6.2)	
Saline, sodium bicarbonate and NAC, *n* (%)	26 (6.2)	11 (5.2)	15 (7.2)	
Procedural and angiographic characteristics				
Mehran 2.0 score (pre-procedural)	8 (7, 10)	8 (6, 10)	9 (7, 10)	.127
Primary PCI, *n* (%)	192 (45.7)	94 (44.5)	98 (46.9)	.630
Radial access, *n* (%)	219 (52.1)	108 (51.2)	111 (53.1)	.693
Femoral access, *n* (%)	201 (47.9)	103 (48.8)	98 (46.9)	
Use of intra-aortic balloon pump, *n* (%)	35 (8.3)	17 (8.1)	18 (8.6)	.837
Contrast volume (mL), median (IQR)	150 (130, 180)	150 (130, 180)	150 (130, 180)	.613
Inotropic support during/post-PCI, *n* (%)	78 (18.6)	41 (19.4)	37 (17.7)	.649

Values are expressed as median (IQR) or *n* (%).

^a^NGAL testing was performed in a subset of 261 study participants.

ACEi/ARB, angiotensin-converting enzyme/angiotensin receptor blocker; BMI, body mass index; DBP, diastolic BP; Hb, hemoglobin; NSTEMI, non-STEMI; CABG, coronary artery bypass grafting; RIPC, remote ischemic preconditioning; SGLT2i, sodium-glucose transporter-2 inhibitor; SBP, systolic BP.

RIPC and sham intervention were conducted as per protocol. The median number of cycles administered was 4 (range 1–7), and the median time interval between completion of the final cycle of RIPC/sham conditioning and the beginning of PCI was 5 [interquartile range (IQR) 0–12] min. There was no difference between the two groups (*P* = .764 and .498, respectively). A total of 51 patients (12.1%) received fewer than four cycles of RIPC/sham conditioning because they were taken for PCI earlier than anticipated; of these, 31 (7.4%) received three cycles, 18 (4.3%) received two cycles and 2 (0.5%) received only a single cycle. Among these 51 patients, 29 (56.9%) belonged to the RIPC group and 22 (43.1%) to the sham group (*P* = .780). Conversely, 75 patients (17.9%) received additional cycles due to unforeseen delays in PCI, with a similar number of patients in both groups receiving additional cycles (*P* = .740). The procedure was well-tolerated, with a median pain score of 3 (IQR 2–4), with no significant difference between the two groups (*P* = .263). There was no reduction in the duration or number of RIPC cycles due to pain. No RIPC-related adverse events were noted.

There was no significant difference between the two groups in terms of PCI characteristics, including the volume of iodinated contrast used (Table 1).

Serum creatinine was assessed at 24- and 48-h post-PCI. A total of 48 (11.4%) developed CA-AKI with stage 1 AKI in 43 (10.2%), stage 2 AKI in 3 (0.7%) and stage 3 AKI in 2 (0.5%). Two patients (0.5%) required dialysis support. In-hospital mortality occurred in 8 (1.9%) and 30-day MACE in 11 (2.6%) patients.

Compared with the sham conditioning group, the incidence of CA-AKI was significantly lower in the RIPC group (8.1% vs 15.0%, RR 0.54, 95% CI 0.31–0.94; *P* = .027, number needed to treat (NNT) 14.5, 95% CI 7.79–139.01). However, there was no significant difference between the delta creatinine of both groups (*P* = .158). The need for renal replacement therapy, in-hospital mortality and 30-day MACE rates was also similar in both groups (Table 2).

**Table 2:** Comparison of study outcomes in both groups.

Outcome	Total (*n* = 420)	RIPC (*n* = 211)	Sham conditioning (*n* = 209)	RR (95% CI)	*P*-value
Primary outcome					
CA-AKI, *n* (%)	48 (11.4)	17 (8.1)	31 (15.0)	0.54 (0.31–0.94)	**.027**
Secondary outcomes					
Delta creatinine	–0.03 (–0.17 to 0.12)	–0.04 (–0.18 to 0.10)	–0.02 (–0.15 to 0.15)		.158
Need for RRT, *n* (%)	2 (0.5)	1 (0.5)	1 (0.5)	0.99 (0.06–15.73)	.995
In-hospital mortality, *n* (%)	8 (1.9)	3 (1.4)	5 (2.4)	0.59 (0.14–2.46)	.467
30-day MACE, *n* (%)	11 (2.6)	4 (1.9)	7 (3.4)	0.57 (0.17–1.90)	.351
Subclinical AKI, *n* (%)^a^	100 (38.3)	47 (36.2)	53 (40.5)	0.89 (0.66–1.22)	.158
Delta 2-h NGAL^a^	6.0 (–34.5, 41.7)	2.3 (–36.7, 37.3)	8.5 (–28.0, 42.3)		.594
Delta 6-h NGAL^a^	12.3 (–31.2, 50.8)	12.3 (–32.5, 47.2)	13.8 (–28.4, 59.3)		.405

Values are expressed as median (IQR) or *n* (%).

^a^Note that the frequencies/percentages or median (IQR) in this row are for the subset of participants in which NGAL testing was performed (*n* = 261).

RRT, renal replacement therapy.

P-value < 0.05 (significance threshold) highlighted in bold.

Analysis for baseline, 2- and 4-h NGAL was conducted in stored serum samples of 261 participants. The baseline characteristics of this subset are tabulated in Table 3. The median NGAL values were 95.6 (58.3, 146.0), 104 (64.0–166.0) and 106 (68.1–158.0), respectively, at baseline, 2-h and at 6-h (*P* = .032). Subclinical AKI occurred in 100 (38.3%) of these 261 participants. The median 2-h NGAL was 94.5 (58.4, 169.0) vs 106.0 (69.7, 155.0) in the RIPC and sham conditioning group, respectively (*P* = .616), while the median 6-h NGAL was 94.5 (60.2, 145.0) in the RIPC group vs 119.0 (74.8, 172.0) in the sham conditioning group (*P* = .032). There was no difference in the proportion of subclinical AKI between the two groups (36.2% vs 40.5%; *P* = .158). Other outcomes are tabulated in Table 2.

**Table 3:** Baseline characteristics of the study subset with NGAL values.^a^

	Study subset with NGAL values (*N* = 261)	RIPC group (*n* = 130)	Sham conditioning group (*n* = 131)	*P*-value
Demographic profile				
Age, years	66.0 (59.0, 73.0)	65.0 (59.0, 71.8)	67.0 (58.5, 73.5)	.398
Male, *n* (%)	193 (73.9)	98 (75.4)	95 (72.5)	.598
Diabetes mellitus, *n* (%)	164 (62.8)	74 (56.9)	90 (68.7)	.049
Hypertension, *n* (%)	167 (63.9)	83 (63.8)	84 (64.1)	.963
Dyslipidemia, *n* (%)	48 (18.4)	27 (20.8)	21 (16.0)	.323
Smoking, *n* (%)	46 (17.6)	24 (18.5)	22 (16.8)	.228
Previous PCI/CABG, *n* (%)	45 (17.2)	21 (16.2)	24 (18.3)	.643
Vitals and anthropometry				
SBP, mmHg	130 (120, 140)	130 (120, 140)	130 (120, 140)	.127
DBP, mmHg	80 (70, 90)	80 (70, 90)	80 (70, 87)	.376
BMI, kg/m^2^	24.9 (23.0, 26.6)	24.7 (22.6, 26.6)	25.1 (23.5, 26.9)	.484
Clinical presentation, *n* (%)				
STEMI	137 (52.5)	65 (50.5)	72 (55.0)	.090
NSTEMI	76 (29.1)	33 (25.4)	43 (32.8)	
Unstable angina	36 (13.8)	23 (17.7)	13 (9.9)	
Stable angina	2 (0.8)	2 (1.5)	0 (0.0)	
Others	10 (3.8)	7 (5.4)	3 (2.3)	
Laboratory values				
Hb, g/dL	12.8 (11.2, 14.3)	12.9 (11.1, 14.6)	12.6 (11.2, 14.2)	.562
HbA1c, %	6.5 (5.7, 8.3)	6.4 (5.7, 8.0)	6.7 (5.8, 8.4)	.236
Serum creatinine, mg/dL	1.3 (1.0, 1.5)	1.2 (1.0, 1.5)	1.3 (1.0, 1.5)	.072
eGFR, mL/min/1.73 m^2^	55 (44, 75.3)	57 (46.5, 78.5)	54 (42, 71)	.135
Albumin, g/dL	4.2 (3.9, 4.5)	4.2 (3.9, 4.5)	4.2 (3.8, 4.5)	.271
Serum NGAL, ng/mL	95.6 (58.3, 146.0)	88.0 (58.0, 132.0)	103.0 (58.5, 148.0)	.293
Medications, *n* (%)				
ACEi/ARB	18 (6.9)	11 (8.5)	7 (5.3)	.320
SGLT2i	54 (21.5)	26 (21.0)	28 (22.0)	.835
Insulin	33 (12.6)	17 (13.1)	16 (12.2)	.834
Statin	251 (96.9)	125 (96.9)	126 (96.9)	.991
CA-AKI prophylaxis, *n* (%)				
Saline only	162 (62.1)	79 (60.8)	83 (63.4)	
Saline and sodium bicarbonate	46 (17.6)	26 (20.0)	20 (15.3)	.675
Saline and NAC	27 (10.3)	14 (10.8)	13 (9.9)	
Saline, sodium bicarbonate and NAC	26 (10.0)	11 (8.5)	15 (11.5)	
Procedural and angiographic characteristics				
Mehran 2.0 score (pre-procedural)	8 (7, 10)	8 (6, 10)	9 (7, 10)	.127
Primary PCI, *n* (%)	78 (56.9)	36 (55.4)	42 (58.3)	.728
Radial access, *n* (%)	138 (52.9)	67 (51.5)	71 (54.2)	.667
Femoral access, *n* (%)	123 (47.1)	63 (48.5)	60 (45.8)	
Use of intra-aortic balloon pump, *n* (%)	16 (6.1)	7 (5.4)	9 (6.9)	.617
Contrast volume (mL), median (IQR)	150 (130, 180)	150 (123, 188)	150 (130, 180)	.373

Values are expressed as median (IQR) or *n* (%).

^a^NGAL testing could not be done in all study participants due to insufficient funding.

ACEi/ARB, angiotensin-converting enzyme/angiotensin receptor blocker; BMI, body mass index; DBP, diastolic BP; Hb, hemoglobin; NSTEMI, non-STEMI; CABG, coronary artery bypass grafting; SGLT2i, sodium-glucose transporter-2 inhibitor; SBP, systolic BP.

### Additional analysis

In addition to the statistical analyses pre-specified in the protocol, we conducted a per-protocol analysis, an adjusted analysis to account for baseline imbalances, sensitivity analyses and subgroup analysis to assess for effect modification. We also assessed the impact of RIPC on 30-day kidney outcomes. The results of these analyses are presented in the Supplementary Appendix.

## DISCUSSION

This study included patients at high risk of CA-AKI who were undergoing either elective or primary PCI and found that RIPC significantly reduced the occurrence of CA-AKI (RR 0.54, 95% CI 0.31–0.94, NNT 14.5; *P* = .027). RIPC was well-tolerated, and no procedure-related adverse events were noted. This is a simple bedside procedure that can be performed by trained nursing personnel, and so can be easily implemented in low-resource settings and even in patients awaiting primary PCI, provided it does not delay revascularization. The mechanism of nephroprotection afforded by RIPC is unknown; however, a humoral basis has been postulated wherein an organ subjected to ischemic conditioning releases humoral factors like adenosine or bradykinin, which subsequently protect the remote organ [8, 25–27]. Anti-inflammatory, anti-antioxidant and neurogenic pathways of effect have also been described [8, 25].

Our results align with a few previous RCTs that have shown that RIPC may confer kidney protective effects in patients receiving intravenous or intraarterial iodinated contrast, though the evidence to date remains conflicting. The Renal Protection trial, which included 100 patients with impaired kidney function (serum creatinine >1.4 mg/dL or eGFR <60 mL/min/1.73 m^2^) undergoing coronary angiography (with or without PCI), showed an overwhelming benefit of RIPC in reducing CA-AKI (12% vs 40%; *P* = .002) and the composite endpoint of death, hospitalization or hemodialysis at 6 weeks (16% vs 38%; *P* = .018) [11]. Notably, a higher number of patients in the control group underwent PCI, resulting in higher contrast volumes that could have led to more number of CA-AKI events, compared with that observed in the RIPC group. The European and Chinese Cardiac and Renal Remote Ischemic Preconditioning Study (EURO-CRIPS) with 223 patients undergoing elective PCI also found that RIPC significantly reduced CA-AKI incidence (12.1% vs 26.1%; *P* = .01) [12]. More recently, the Biochemical and Reno-Protective Effects of Remote Ischemic Preconditioning on Contrast-Induced Kidney Disease (BRICK) trial, which included high-risk patients undergoing coronary angiography, suggested that RIPC may reduce AKI incidence by nearly 50% [28]. RIPC administered 24 h prior to coronary angiography (delayed RIPC) was also found to have a nephroprotective effect in a large multicenter study [29]. In contrast, other trials have not demonstrated consistent benefits. Menting *et al*. found that the change in serum creatinine from baseline to 48–72 h of contrast administration (delta creatinine) was similar in the RIPC and control groups, although in those with a Mehran risk score ≥11, RIPC led to a significant reduction in delta creatinine [13]. PRotection against contrast mEdium-induced nephropaPAthy in patients at Risk undErgoing coronary angiography (PREPARE) trial and/or PCI, found that RIPC impacted neither the incidence of CA-AKI (3.8% vs 5.1%; *P* = .74) nor clinical outcomes at 12-month follow-up [30]. Other randomized studies have also failed to demonstrate a clear benefit [14, 31, 32]. While systematic reviews and meta-analysis indicate that RIPC is associated with a lower risk of CA-AKI in patients undergoing coronary angiography or PCI, many of the included studies were small and assessed as having an unclear or high risk of bias [33, 34]. Moreover, many of the available studies have included patients receiving intravenous contrast and/or those undergoing coronary angiography without PCI leading to heterogeneity. Previous studies have also excluded patients undergoing primary PCI, although a pilot study of 125 STEMI patients undergoing primary PCI found that RIPC significantly reduced CA-AKI (odds ratio 0.18, 95% CI 0.05–0.64; *P* = .008) [10]. In our study, approximately 45% of the study cohort comprised of those who underwent primary PCI, which demonstrates the feasibility and safety of RIPC even in this setting. The nephroprotective potential of RIPC has also been studied in the setting of cardiac surgery, and the evidence is conflicting. While two large, randomized trials found no kidney benefit from RIPC in patients undergoing cardiac surgery, results from smaller studies involving high-risk populations have reported a significant reduction in AKI events [29, 35–37].

It has been reported that 15%–20% patients with normal creatinine may have elevated levels of biomarkers like NGAL and such “biomarker-positive, creatinine-negative” patients have poorer short- and long-term outcomes, compared with their “biomarker-negative, creatinine-negative” counterparts [23, 38–40]. These observations have led to the emergence of a new entity called subclinical AKI, a condition where there is evidence of structural renal injury as indicated by high biomarker levels, but without clinical AKI [41]. The impact of RIPC on subclinical AKI remains a relatively underexplored area. While there is currently no universally accepted definition of subclinical AKI, we adopted a criterion based on an increase of ≥25% in plasma NGAL from baseline, as described in two previous studies [23, 24]. This approach of using relative changes in NGAL, rather than an absolute threshold, may help minimize confounding due to interindividual variability in baseline levels and may better reflect an acute change attributable to contrast administration. In our study, among the subset of patients in whom NGAL testing was performed, 38.3% were found to have subclinical AKI. Although subclinical AKI was numerically lower in the RIPC group (36.2% vs 40.5%), the difference was not statistically significant. The present study was underpowered (post-hoc power: 10.6%) for this study outcome since NGAL testing could not be performed in all study participants. Further studies with larger sample sizes are therefore warranted to definitively assess the effect of RIPC on subclinical AKI. A meta-analysis of three RCTs found that NGAL levels post-PCI/coronary angiography were lower in those undergoing RIPC [33]. Our study too found that the median 6-h NGAL was significantly lower in the RIPC group compared with the sham conditioning group (94.5 vs 119.0; *P* = .032), although there was no statistically significant difference in the 2-h NGAL values.

To the best of our knowledge, this is one of the largest RCTs of RIPC for CA-AKI prevention in the PCI population to date. The inclusion of PCI patients only, specifically, an enriched cohort of PCI patients at high risk of CA-AKI, is another strength of this study. Furthermore, this study included even primary PCI cases, a group in which evidence for RIPC in CA-AKI prevention has been hitherto lacking. However, this study has a few limitations. Firstly, this was a single-center study which may limit the generalizability of our results. Secondly, CA-AKI was defined based on creatinine criteria alone since urine output recordings are often unreliable, and this may have led to an underestimation of CA-AKI incidence in this study. In addition, creatinine is a late and insensitive biomarker and can be influenced by several factors, including possibly high-pressure BP cuff inflation itself; therefore, in the absence of other biomarkers like cystatin C, it remains uncertain whether the observed changes in creatinine truly reflected kidney injury. Although all patients received iso-osmolar contrast and intravenous hydration, the fluid regimen and use of adjunctive nephroprotective agents such as NAC or sodium bicarbonate were individualized based on clinical judgment to reflect real-world practice, rather than strictly protocolized. While efforts were made to minimize procedural risks, other potential contributors to AKI, such as atheroembolic kidney disease, could not be definitively excluded. Therefore, we have used the term CA-AKI rather than contrast-induced AKI to reflect the multifactorial pathogenesis of AKI in this setting [7]. Additionally, though all secondary outcomes were pre-specified, no statistical adjustment was made for multiple comparisons. Since none of these outcomes reached statistical significance, the risk of false-positive findings is minimal; nonetheless, these findings should be interpreted in an exploratory context. Furthermore, due to funding constraints, NGAL testing to detect subclinical AKI could not be performed for all study participants, resulting in reduced power for this outcome. Lastly, we did not assess long-term effects of RIPC on kidney function and patient outcomes.

An important caveat is that while RIPC reduced the incidence of CA-AKI in this study, 89.5% of the observed events were stage 1 AKI only. While the clinical relevance of preventing milder forms of AKI may be debatable, it is important to keep in mind that even stage 1 AKI may be associated with adverse long-term outcomes [42]. Another notable takeaway from this study is that the incidence of CA-AKI was only 11.4%, which is much less than what one would expect in this selected cohort of high-risk patients. This illustrates the point that CA-AKI may not be as common as previously believed and that in contemporary practice, fear of worsening kidney function should not lead to withholding or delay of life-saving procedures such as PCI (“renalism”) [43].

## CONCLUSIONS

RIPC offers a safe and feasible approach for CA-AKI prevention in high-risk patients undergoing PCI. Further studies are needed to understand the mechanism of nephroprotection and its impact on subclinical AKI.

## Supplementary Material

sfaf342_Supplemental_File

## Data Availability

The data that support the findings of this study are available from the corresponding author upon reasonable request.
