# Supplementary material for: Remote ischemic preconditioning for prevention of contrast-associated acute kidney injury following percutaneous coronary intervention: a randomized controlled trial
Source: Clin Kidney J. 2025 Nov 7;18(12):sfaf342. doi: 10.1093/ckj/sfaf342 (PMC12665983; doi:10.1093/ckj/sfaf342)
Supplement: sfaf342_Supplemental_File [file sfaf342_supplemental_file.docx]

Supplementary Appendix

Contents

[1. Per Protocol Analysis 1](#_Toc207229420)

[2. Adjusted Analysis 1](#_Toc207229421)

[3. Sensitivity Analyses 1](#_Toc207229422)

[4. Subgroup Analysis 2](#_Toc207229423)

[5. Additional Outcomes 2](#_Toc207229424)

[6. Details of Patients Who Received Dialysis 2](#_Toc207229425)

## 1. Per Protocol Analysis

In the per-protocol analysis including only participants who received exactly four cycles of RIPC (n=294), the incidence of CA-AKI was 8.2% in the RIPC group (12/146) compared with 15.5% in the control group (23/148) (RR 0.53, 95% CI: 0.27–1.02; p= 0.053). Although the difference did not reach statistical significance, likely due to reduced sample size, the effect direction and magnitude were consistent with the primary analysis.

## 2. Adjusted Analysis

An adjusted analysis was performed using log-binomial regression to estimate adjusted RRs, using diabetes mellitus and baseline creatinine as covariates based on observed baseline imbalances between the RIPC and control groups (p < 0.10). The intervention group (RIPC) had a lower risk of contrast-associated AKI compared to the control group (adjusted RR 0.50, 95% CI 0.26–0.97; p = 0.041), consistent in direction and magnitude with the unadjusted analysis.

## Sensitivity Analyses

- 1. Using Original Eligibility Criteria Before Protocol Amendment

In the sensitivity analysis restricted to participants meeting the original eligibility criteria (n=274), the incidence of CA-AKI remained numerically lower in the RIPC group (10.4%) compared with the control group (18.0%) (RR 0.58, 95% CI: 0.31–1.06; p=0.071). Although the difference did not reach statistical significance, likely due to reduced sample size, the effect direction and magnitude were consistent with the primary analysis.

- 1. Using KDIGO Definition for CA-AKI

Using the KDIGO definition (increase in serum creatinine by ≥0.3 mg/dL or ≥1.5 times of baseline values within 7 days), the incidence of CA-AKI was 8.5% in the RIPC group (18/211) compared with 16.3% in the control group (34/209), corresponding to an RR of 0.52 (95% CI:0.31- 0.90; p = 0.019), which was consistent with the primary analysis.

## Subgroup Analysis

Subgroup analyses stratified by contrast volume (low vs high, using the median as cut-off) and diabetes status were performed to assess for effect modification. No statistically significant interactions were observed between treatment allocation and either diabetes status (p = 0.074) or contrast volume (p = 0.162). As these analyses were not prespecified, they should be considered exploratory.

## Additional Outcomes

In addition to the prespecified endpoints, we assessed major adverse kidney events at 30 days (MAKE30) and serum creatinine at 30 days.

MAKE30 was defined as a composite of renal replacement therapy (RRT), all-cause mortality, or persistent kidney dysfunction (final serum creatinine value before hospital discharge ≥200% of baseline), evaluated at day 30.

MAKE30 occurred in 5 of 211 patients (2.4%) in the RIPC arm and 8 of 209 patients (3.8%) in the control arm (p = 0.388). Median serum creatinine at day 30 was 1.16 mg/dL (IQR 0.94–1.44) in the RIPC arm and 1.22 mg/dL (IQR 1.01–1.66) in the control arm, with no significant difference between groups (p = 0.097).

## Details of Patients Who Received Dialysis

Details of study participants who needed dialysis following PCI are mentioned in Supplementary table 1.
